# Passive movement and active exercise for very young infants with congenital heart disease: a study protocol for a randomized controlled trial

**DOI:** 10.1186/s13063-015-0816-9

**Published:** 2015-06-30

**Authors:** Qing Du, Xuan Zhou, Xueqiang Wang, Sun Chen, Xiaoyan Yang, Nan Chen, Juping Liang, Weiwei Deng, Kun Sun

**Affiliations:** Department of Rehabilitation Medicine, Xin Hua Hospital Affiliated to Shanghai Jiao Tong University School of Medicine, Shanghai, China; Sport Medicine and Rehabilitation Centre, Shanghai University of Sport, Shanghai, China; Department of Pediatric Cardiology, Xin Hua Hospital Affiliated to Shanghai Jiao Tong University School of Medicine, No. 1665 Kongjiang Road, Shanghai, China; Department of Kinesiology, Shanghai University of Sport, Shanghai, China

**Keywords:** Congenital heart disease, Physical therapy, Exercise therapy, Randomized controlled trial, Rehabilitation

## Abstract

**Background:**

Delayed motor development is reported in patients with congenital heart disease (CHD). Exercise is widely used to facilitate motor development and improve motor ability. Exercise for adolescents and adults with CHD has been extensively studied. However, the evidence of exercise for infants with CHD is sparse. This study aims to identify the effect of passive movement and active exercise on motor development within very young CHD infants with cardiac catheterization.

**Methods/Design:**

A prospective and randomized controlled trial will be conducted in very young CHD infants with cardiac catheterization. A total of 147 infants with CHD will be randomized by a 1:1:1 allocation ratio by computer to an exercise intervention group, a home-based intervention group and a control group. The exercise intervention group will receive passive movement and active exercise from experienced physiotherapists in pediatrics three times a week for 12 weeks. The home-based intervention group will receive passive movement and active exercise from their parents or caregivers at home three times a week for 12 weeks. The control group will receive follow up only. The follow-up duration is 20 months. The primary outcome measures are the motor quotient measured by the Peabody Developmental Motor Scales-II. The secondary outcome measures are the Ross score, ventricular function, bone quality, body length, weight, head circumference, upper arm circumference, and adverse events.

**Discussion:**

This study has several important features, including the randomization process, the long follow-up duration, the control group, and the large sample size. The aim of this study is to determine whether 12-week passive movement and active exercise promotes motor development and produces other beneficial effects for very young CHD infants with cardiac catheterization. Therefore, this study will contribute new knowledge regarding the rehabilitation program in very young CHD infants with cardiac catheterization.

**Trial registration:**

Current Controlled Trials ChiCTR-IOR-15005909 (January 31, 2015).

## Background

Congenital heart disease (CHD) is common in infants [1]. The prevalence of CHD was 10.8-26.6‰ [2–4]. With the improvement of echocardiography and medical care, more patients with CHD were underwent cardiac catheterization than ever before. Because of the high success rate, short hospital stay, low morbidity and mortality, cardiac catheterization of CHD is favored over surgery. Cardiac catheterizations have been used to treat neonates with critical congenital heart disease, such as valvar stenosis or atresia [5]. However, patients with CHD frequently exhibit delayed neurodevelopmental function, decreased exercise capacity, impaired exercise performance and lower health-related quality of life.

Pronounced delayed neurodevelopmental function has been observed in children with CHD [6, 7], especially those with cyanotic CHD [7] or catheterization [8] or heart surgery performed with circulatory arrest [9]. Lower exercise function [10, 11], dysfunction in speech and language [12], poor cognitive function [13, 14] and behavior difficulties [8] were also reported in preschool and school-age patients with CHD. The causes of delayed neurodevelopmental function may include hemodynamic defects, long-standing hypoxemia [7], long hospital stay and physical inactivity (for example, prolonged time in strollers and walkers). Inadequate exercise for patients with CHD may lead to cardiovascular complications. Therefore, infants with CHD require a large amount of physical activity to master movement control.

The Cardiac Rehabilitation Program has been studied in adolescents and adults with CHD [15–17]. Exercise is the core component of the cardiac rehabilitation program and is safe, feasible and beneficial. Past studies have documented that exercise increases exercise capacity [10, 18–20], HR recovery following peak exercise [21], respiratory muscle oxygenation and health-related quality of life [15, 16] in adolescents and adults with CHD. In contrast, the use of the cardiac rehabilitation program and clinical research in pediatric populations have been limited [22]. There is only one study of preschool children with CHD, which suggests that the short low-dose intervention program can improve motor ability and delayed motor development [23]. The optimal cardiac rehabilitation program of this unique population is still unknown and needs to be developed.

Performing exercises in infants with CHD remains challenging. To date, there have been few rehabilitation interventions that target infants with CHD. The effect of exercise on motor development in infants with CHD is unknown. Therefore, it is important to identify passive movement and active exercise to facilitate motor development within this population. This paper describes the rationale, design and methodology of a randomized, controlled study being conducted in China.

### Methods/Design

A prospective and randomized controlled trial involving passive movement and active exercise in very young CHD infants with cardiac catheterization will be performed. The aim of the study is to determine:

1. whether passive movement and active exercise beginning at 1 month of corrected age (CA) in infants with CHD improves motor development at 24 months of CA compared with control subjects,
2. the potential benefits of exercise on growth, heart function and bone quality of very young infants with CHD, and
3. the potential adverse effects related to passive movement and active exercise.

## Study design

The CHD patients with cardiac catheterization will be recruited from and investigated at the Xin Hua Hospital affiliated to the Shanghai Jiao Tong University School of Medicine, Shanghai City, China.

Prior to inclusion, the following information will be obtained for all patients: demographic data (for example, gestational age at birth, birth weight, CA, and gender), medical history (for example, the duration of intensive care unit and hospital stay, and age of cardiac catheterization), heart defect (for example, defect type and size), and type of feeding (for example, breast or artificial feeding). Each patient’s parents will be asked to sign an informed consent form before the patient can participate in the study.

**Fig. 1:**
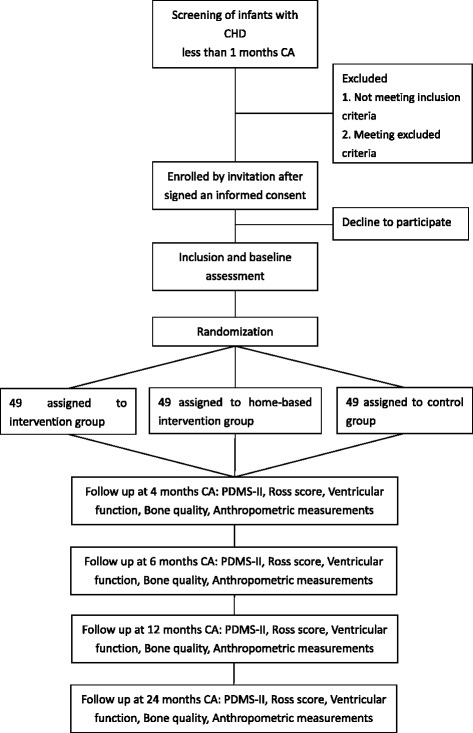
Flowchart. CHD indicates congenital heart disease; *CA* corrected age, *PDMS-II* Peabody Developmental Motor Scales-II

Eligible participants will be randomized by a 1:1:1 allocation ratio by computer either to the exercise intervention group, in which they will receive passive movement and active exercise to promote motor development; the home-based intervention group, in which they will receive passive movement and active exercise from parents or caregivers at home; or the control group, in which they receive follow up only (Fig. 1). The total study duration will be 23 months, which consists of a 3-month intervention and 20-month follow-up period without intervention. All patients will receive the exercise intervention, the home-based intervention, or the control conditions as allocated. All patients will be assessed at 1, 4, 6, 12 and 24 months CA.

If the pediatrician evaluating the patient prescribes additional physiotherapy, the patient will receive physiotherapy. The pediatrician will record information if the participant receives other physiotherapy.

## Participants

CHD patients with cardiac catheterization are eligible for the study. Patients who meet the following inclusion criteria will be enrolled:

1. infants with CHD,
2. less than 1 month CA,
3. with a modified Ross score of less than 3 points,
4. availability 5 days a week over a period of 12 weeks,
5. for whom informed parental consent has been provided.

Patients who have any of the following exclusion criteria will be excluded:

1. previous cardiac operation history;
2. a genetic syndrome, respiratory failure, neurological disorder, or arrhythmia;
3. heart failure with a modified Ross score of 3 points or more;
4. significant hemodynamic defects to make exercises inadvisable; or
5. other congenital malformations.

## Withdrawal management

The patients with CHD who have any of the following conditions will be allowed or will be asked to withdraw from the study:

1. The patient’s parents require withdrawal.
2. The patient develops heart failure, or respiratory failure, or other serious diseases.
3. The exercises lead to any adverse effects.

## Ethical considerations

The ethics committee of Xin Hua Hospital affiliated to the Shanghai Jiao Tong University School of Medicine approved the study protocol (Approval No. XHEC-D-2015-002). The ethics committee certified that this study did not raise any issues of patients’ risk. The ethics committee also certified that the study was in accordance with the Declaration of Helsinki and that the study was conducted without ethics problems. The study protocol was registered at www.chictr.org.cn, number identifier: ChiCTR-IOR-15005909.

## Recruitment process

Enrollment will take place at the pediatric cardiovascular clinic of the Xin Hua Hospital. A pediatrician will evaluate all of the eligible patients. Written informed consent must be signed by the patients’ parents prior to enrollment. Then, we will perform the baseline assessment.

## Randomization process

The participants are randomly assigned to each group after the baseline assessment, when the demographic data are collected. We use concealed randomization assignment and an adequate computer-generated assignment sequence to avoid selection bias. Thus, neither investigators nor the participants’ parents can influence which group the patients are assigned to.

## Intervention group

Exercise will be provided for a 25-min period per day for 5 days a week over a period of 12 weeks, 1 h after feeding by experienced pediatric physiotherapists. All infants in this group will receive exercise in supine, prone, side-lying, and supported-sitting positions. The specific exercise protocol is selected based on previous studies [24–30]. The exercise protocol consists of 25 minutes of movements for very young infants, including passive range-of-motion exercise, feet and hands reaching, and exercises in prone, side-lying, and supported-sitting positions. Table 1 presents the details about the exercise protocol. Experienced pediatric physiotherapists will perform the exercise intervention. If the infant begins to cry, the exercises will be stopped. Each infant will receive treatment from multiple physiotherapists to ensure that the treatment effects are the result of the treatment protocol and not any particular provider. Each infant will receive exercise in a random beginning position and sequence of movements within each position. The parents or caregivers will be asked to record the level of compliance with the exercises each day. The level of compliance will be scored using the following categories [31]: tolerated movement well, tolerated movement moderately well, refused to participate, or is not present in the hospital.

**Table 1:** The exercise protocol for very young infants with CHD

Positions	Movements	Each time (s)	Repetitions (n)	Total time (s)
Supine	Passive range-of-motion exercise			
	Flexing and extending each hand at the wrist	5	10	50
	Flexing and extending each arm at the elbow	5	10	50
	Flexing and extending each arm at the shoulder	5	10	50
	Flexing and extending each foot at the ankle	5	10	50
	Flexing and extending each leg at the knee	5	10	50
	Flexing and extending each leg at the hip	5	10	50
	Feet reaching to contact a midline toy			
	Holding the infant’s leg and directing the foot toward the toy in midline	30	2	60
	Holding a stationary toy in midline within the infant’s visual field, and leading the foot toward the toy for a few seconds to allow the infant to contact the toy spontaneously. If the infant do not contact the toy spontaneously, the physiotherapist performs tactile stimulation with the toy to the infant’s foot	30	2	60
	Holding an infant’s hip at 90 degrees, holding a stationary toy within the infant’s visual field, and encouraging the foot to contact the toy	30	2	60
	Hand reaching to contact a midline toy			
	Holding the infant’s forearm and directing the hand toward the toy in midline	30	2	60
	Performing tactile stimulation with the toy on one upper limb of the infant, taking the toy to the midline, and waiting a few seconds to allow the infant to contact the toy with the hand spontaneously	30	2	60
	Holding a stationary toy in the midline within the infant’s visual field, and leading the hand toward the toy for a few seconds to allow the infant to spontaneously contact the toy. If the infant does not spontaneously contact the toy, the physiotherapist performs tactile stimulation with the toy in the infant’s hand	30	2	60
Prone	Prone on a ball: use elbows to support the weight and raise the head	30	4	120
	Hand reaching to contact a toy slightly out of reach	30	4	120
Side-lying	Anterior-posterior rocking: place hands on hips and on the shoulders and head; slowly rock forward and backward	6	10	60
	Lower trunk rotating: placing hands between lower extremities to control pelvis; slowly rotate the pelvis on the upper trunk anteriorly and posteriorly	6	10	60
Supported-sitting	Displacing the trunk slowly in anterior, posterior, and lateral directions, 30 degrees from a vertical position. Place back upright to 20 degrees of a vertical position. Head and upper trunk supported throughout entire displacement	6	10	60
	Rotating the upper body slowly to each side with the head and upper extremities supported; approximately 45 degrees of rotation	6	10	60
	Supporting infant 20 degrees from a vertical position; placing index fingers on side of head to control bobbing. Briefly removing head support to encourage head control.	6	10	60

### Exercise in the supine position

#### Passive range-of-motion exercise

Previous studies have proven that passive range-of-motion exercise with gentle joint compressions is beneficial for weight gain [25, 32] and bone development [26, 33–36] in preterm infants. Consequently, effective passive exercise may be needed in infants with CHD. Each infant will receive passive range-of-motion exercises with gentle joint compressions at the end of each movement. We will perform 10 repetitions of passive range-of-motion exercise on the wrists, elbows, shoulders, ankles, knees, and hips. Each joint flexion/extension motion lasts for 5 s, for a total period of 10 min.

#### Feet and hand reaching

Goal-directed reaching provides an early intervention strategy to encourage movement in very young infants. Studies have showed that feet and hand reaching training programs were beneficial for the preterm population [27, 28, 37, 38]. Feet and hand reaching using a malleable rubber toy are designed to improve the ability of infants to reach for toys with their feet or hands and promotes the dissociation of the joints. Both feet and hand reaching consists of toy-oriented reaching in three movements by a physiotherapist, as described in a previous study [27, 38]. We will perform 2 repetitions. Each movement lasts for approximately 30 s, for a total period of 6 min.

### Exercise in the prone position

The prone position is a very important position for infants developing motor development. Young infants are able to take advantage of the prone position to advance their early motor development [39]. The exercises in prone position are designed to develop antigravity movements of the extremities, head control, weight bearing on arm muscles, and strength for sitting. The exercises in the prone position consist of two movements (Table 1) as described in a previous study [29]. Each movement is repeated four times. Each movement lasts for approximately 30 s, for a total period of 4 min.

### Exercise in the side-lying position

Each infant will receive exercises in the side-lying position. Infants will be placed in the side-lying position, in which their hips and knees are flexed to approximately 110 degrees, their heads are slightly flexed forward, and their upper extremities are protracted with their hands placed at the midline. Exercises in the side-lying position are designed to encourage visual awareness and midline orientation of the upper extremities and to promote the development of rolling over. Two movements based on a previous study [30] will be performed in the side-lying position (Table 1). Each movement is repeated ten times. Each movement lasts for approximately 6 s, for a total period of 2 min.

### Exercise in the supported-sitting position

Infants will participate in supported-sitting position exercises. Exercises in the supported-sitting position are designed to promote head and trunk control. Three movements will be performed in the supported-sitting position (Table 1) as described in a previous study [30]. Each movement is repeated ten times. Each movement lasts approximately 6 s, for a total period of 3 min.

## Home-based intervention group

The exercise protocol for the home-based intervention group is the same as that for the intervention group. Exercise intervention will be performed by parents or caregivers. Before starting the home-based intervention, an experienced pediatric physiotherapist will give a detailed explanation of the aims, importance, methods, and procedure of home-based intervention to parents or caregivers. Then, the physiotherapist will teach parents or caregivers the exercises in the four different positions until they perform all the items well. A brochure of home-based intervention and a DVD with exercises video will also be given to them. Parents or caregivers will be asked to record the treatment items, the duration of treatment and the infant’s reaction during the treatment everyday. Physiotherapists will make a phone call once a week to remind them of performing the exercises, and get valuable information about the details of home-based intervention. The level of compliance will be scored using the following categories [31]: tolerated movement well, tolerated movement moderately well, or refused to participate.

## Control group

Each infant in the control group will receive no training. All participants in the trial will receive health education. This includes group counseling focusing on daily activity and diet. The participants will be asked not to participate in any other rehabilitation treatments.

## Outcome measures

The baseline and follow-up evaluations will be performed by experienced pediatric assessors blinded to the group assignments when the patient is at 1, 4, 6, 12 and 24 months CA.

### Primary outcome measure

Motor development will be measured using the Peabody Developmental Motor Scales-II (PDMS-II) via the motor quotient. Motor development is measured at 1, 4, 6, 12 and 24 months CA with the PDMS-II by an assessor blinded to the group assignments. The use of the PDMS-II is recommended [40] and has been widely used as a motor development outcome measure for patients with CHD [41–46]. Both fine and gross motor function can be assessed by the PDMS-II. The PDMS-II is valid for children aged from term to 76 months. The PDMS-2 contains six subtests, including reflexes, stationary, locomotion, object manipulation, grasping and visual-motor integration. The results of the subtests may be used to generate a Gross Motor Quotient, a Fine Motor Quotient and a Total Motor Quotient, which are three global indices of motor development. A previous study demonstrated that the PDMS-II had high test-retest reliability and acceptable responsiveness to intervention effects [47].

### Secondary outcome measures

1. The Ross score will be evaluated using a modified Ross scoring system. The Ross scoring system [48] was first described by Ross [49] for infants. Then, Reithmann et al. [50] and Läer et al. [51] modified the system. The modified Ross scoring system is used in the clinical evaluation of patients with CHD. It consists of diaphoresis, tachypnea, breathing with abdominal retractions, respiratory rate, heart rate, and hepatomegaly. Each variable receives a score of 0, 1, or 2 points according to severity. The range of the total score is from 0 to 12 points with higher values indicating more severe impact. An experienced pediatric cardiologist grades the six variables.
2. Ventricular function will be assessed using echocardiography, which is a useful noninvasive method. In patients with CHD, the measurement of ventricular function using echocardiography is a standard procedure [52]. The left ventricular function is an important variable. The most commonly utilized indices of the left ventricular function are the left ventricular ejection fraction and fractional shortening [48]. Echocardiographic assessment of the patients is performed by an experienced pediatric cardiologist. Echocardiographic assessment of crying children is performed approximately 5 minutes after they become calm.
3. Bone quality will be measured using a quantitative ultrasound machine (Omnisense 7000P, Sunlight Medical Inc., Israel). Quantitative ultrasound measurements provide a safe, non-invasive method for obtaining information on bone quality. The quantitative ultrasound method is designed to measure the speed of sound (SOS) at a peripheral site (such as the distal 1/3 of the radius and middle of the tibia [53]) by axial transmission. The measurement site is defined as the distal 1/3 of the radius. The mean of three measurements of the radius SOS is used for analysis. The z-score is given in terms of the standard deviation from the average of population of same age and gender. The z-score is defined as the difference between the raw score to be standardized and the mean difference divided by the standard deviation (a z-score less than or equal to −2.0 indicates low bone quality and a z-score greater than −2 indicates normal bone quality). The z-score is predictive of bone quality in children [54, 55]. All quantitative ultrasound measurements are performed by the same trained pediatrician.
4. Anthropometric measurements, including body length, weight, head circumference and upper arm circumference, will be measured. Body length is measured without shoes. Weight is measured with light clothing using digital scale (Seca Digital Column Scale and Seca 376 Baby Scale). Head circumference and upper arm circumference are measured with a nonflexible tape.
5. Adverse events related to exercise will be recorded.

### Sample size calculation

We used GPower3.1.9.2 to perform the power calculation. Our primary outcome measure is the motor quotient measured on the PDMS-II. A difference of the motor quotient score among the three groups of 0.5 SD is considered to be clinically significant. Thus, we must recruit 42 infants in each group to have an 80% chance of detecting this difference among the three groups with a significance level of 0.05 (alpha) on two-sided tests. Considering potential attrition, 49 infants in each group will be recruited.

## Statistical analysis

The SPSS 20.0 and Microsoft Excel 2013 software will be used for statistical analyses. Demographic data will be described with descriptive statistics, including the mean ± standard deviation (SD). We will use analysis of covariance to compare the primary and secondary outcomes of the three groups. A general linear model will be used to compute mean outcome values adjusted for diagnosis, gender and socioeconomic status (SES), with the group as the fixed factor and diagnosis, gender and SES as covariates. If patients are lost to follow-up, an intention-to-treat analysis will be performed. Statistical significance is set at a p-value less than 0.05.

## Discussion

According to previous studies, exercise is an effective treatment for adolescents and adults with CHD. However, the evidence of the effects of exercise for CHD infants with cardiac catheterization is sparse. We will perform a prospective randomized-controlled trial of passive movement and active exercise for very young CHD infants with cardiac catheterization. The chosen method seems safe, applicable and feasible to very young infants with CHD. The study may provide evidence that passive movement and active exercise offer an alternative treatment for very young infants with CHD. Passive movement and active exercise may have some beneficial effects for infants with CHD by promoting motor development and growth, and improving heart function and bone quality.

This study has some strengths. First, the follow-up duration in most previous exercise studies typically ranged from 12 weeks [23] to 1 year [20]. In this study, the total study period is 23 months, with an intervention duration of 12 weeks and a follow-up duration (with no active intervention) of 20 months. Second, in this study, the intervention group performs exercises with the assistance of experienced pediatric physiotherapists, meanwhile the home-based intervention group performs exercises with the assistance of parents or caregivers, but the control group receives no training. Therefore, compared to previous studies [18–20, 23], our study could reduce biases. Third, a power calculation is performed for this study. The total number of patients enrolled in the study is 147. Fourth, previous studies have mainly focused on exercise capacity [10, 18–20] and motor development [23]. Heart function and bone quality are rarely analyzed following passive movement and active exercises in patients with CHD. However, there are some limitations in our study. The spontaneous daily activities outside the study exercises are not monitored. Parents or caregivers may alter their infants’ daily activities, which would influence motor development.

## Trial status

Subject recruitment is underway.

## Abbreviations

CHD: Congenital heart disease
CA: Corrected age
PDMS-II: Peabody Developmental Motor Scales-II
SOS: Speed of sound
